# Supplementary material for: Fostering human learning in sequential decision-making: Understanding the role of evaluative feedback
Source: PLoS One. 2024 May 28;19(5):e0303949. doi: 10.1371/journal.pone.0303949 (PMC11132519; doi:10.1371/journal.pone.0303949)
Supplement: Attachment — Submitted filename: Response_to_Reviewers.pdf [file pone.0303949.s001.pdf]

Rei Akaishi
Academic Editor
PLOS ONE

Piyush Gupta
Electrical and Computer Engineering
Michigan State University
East Lansing, MI 48824, USA
email: guptapi1@msu.edu

East Lansing, April 24, 2024

Re: Paper PONE-D-23-36714: *Fostering Human Learning in Sequential Decision-Making: Understanding the Role of Evaluative Feedback*

Dear Professor Akaishi,

Please find enclosed the revision of our manuscript number PONE-D-23-36714, submitted to *PLOS ONE*.

We would like to thank you all for your patience and efforts in reviewing our paper. We appreciate the thoughtful and constructive comments from the reviewers on how to improve the paper. In the following pages, we provide a detailed list of the changes that have been made in the revised version according to the reviewers' observations.

We look forward to hearing from you.

Sincerely yours,

Piyush Gupta
Subir Biswas
Vaibhav Srivastava

Statement of Revision

In the following, we provide a detailed account of all the changes that we have made in the revised version of the paper. We have structured this list in separate blocks, corresponding to the suggestions made by the reviewers. We would like to thank all the reviewers for their thoughtful and constructive remarks. Additionally, we have also updated the bibliography.

Editorial Comments

[E: 1] "The manuscript presents an intriguing integration of methodologies traditionally not combined within a single study. It has been observed that reviewer opinions diverge considerably; however, a closer examination reveals a consensus regarding the fundamental critiques. Specifically, the manuscript draws upon methodologies from distinct academic disciplines, a factor which, when evaluated within the confines of these individual fields, might typically warrant a major revision or outright rejection. This interdisciplinary approach, while innovative, poses significant challenges for evaluation, given that most reviewers possess expertise in a singular discipline. Despite these challenges, the interdisciplinary methodology employed by the authors holds substantial value and represents a meaningful contribution to the field. To enhance the manuscript's impact and accessibility, it is imperative for the authors to revise the manuscript in a manner that renders it comprehensible to a broader audience. Additionally, there is a substantial need for the improvement of the methods employed in the study. In light of these considerations, the editorial decision necessitates a major revision. The authors are encouraged to undertake a comprehensive revision that addresses the aforementioned concerns, with a particular focus on making the manuscript more accessible to readers from various disciplinary backgrounds and enhancing the rigor and clarity of the methodologies employed."

We thank the editor for their time to review our work and their constructive comments to improve our manuscript. In the revised manuscript, we have addressed the reviewers comments and made the manuscript more accessible to readers from various disciplines by including an introduction to reinforcement learning concepts.

Journal Requirements

[E: 1] "1. When submitting your revision, we need you to address these additional requirements. Please ensure that your manuscript meets PLOS ONE's style requirements, including those for file naming. The PLOS ONE style templates can be found at https://journals.plos.org/plosone/s/file?id=wjVg/PLOSOne_formatting_sample_main_body.pdf and https://journals.plos.org/plosone/s/file?id=ba62/PLOSOne_formatting_sample_title_authors_affiliations.pdf."

We are using the Plos One template.

[E: 2] "2. Please note that PLOS ONE has specific guidelines on code sharing for submissions in which author-generated code underpins the findings in the manuscript. In these cases, all author-generated code must be made available without restrictions upon publication of the work. Please review our guidelines at https://journals.plos.org/plosone/s/materials-and-software-sharing#loc-sharing-code and ensure that your code is shared in a way that follows best practice and facilitates reproducibility and reuse."

We have made the code and data accessible at `https://github.com/piyushgupta221/Decision_making_in_ToH`.

[E: 3] "3. Note from Emily Chenette, Editor in Chief of PLOS ONE, and Iain Hrynaszkiewicz, Director of Open Research Solutions at PLOS: Did you know that depositing data in a repository is associated with up to a 25% citation advantage (https://doi.org/10.1371/journal.pone.0230416)? If you've not already done so, consider depositing your raw data in a repository to ensure your work is read, appreciated and cited by the largest possible audience. You'll also earn an Accessible Data icon on your published paper if you deposit your data in any participating repository (https://plos.org/open-science/open-data/#accessible-data)."

We have shared the data which is available at `https://github.com/piyushgupta221/Decision_making_in_ToH`.

[E: 4] "4. Thank you for stating the following financial disclosure: [This work has been supported in part by the NSF awards IIS-1734272 and ECCS-2024649. The analysis portion of this work is supported in part by the ONR award N00014-22-1-2813]. Please state what role the funders took in the study. If the funders had no role, please state: ""The funders had no role in study design, data collection and analysis, decision to publish, or preparation of the manuscript."" If this statement is not correct you must amend it as needed. Please respond by return e-mail so that we can amend your financial disclosure and competing interests on your behalf."

We have requested an update to the financial disclosure as follows:

[This work has been supported in part by the NSF awards IIS-1734272 and ECCS-2024649. The analysis portion of this work is supported in part by the ONR award N00014-22-1-2813. The funders had no role in study design, data collection and analysis, decision to publish, or preparation of the manuscript.]

[E: 5] "5. Thank you for stating the following in the Acknowledgments Section of your manuscript: [This work has been supported in part by the NSF awards IIS-1734272 and ECCS-2024649. The analysis portion of this work is supported in part by the ONR award N00014-22-1-2813.] We note that you have provided funding information that is not currently declared in your Funding Statement. However, funding information should not appear in the Acknowledgments section or other areas of your manuscript. We will only publish funding information present in the Funding Statement section of the online submission form. Please remove any funding-related text from the manuscript and let us know how you would like to update your Funding Statement. Currently, your Funding Statement reads as follows: [This work has been supported in part by the NSF awards IIS-1734272 and ECCS-2024649. The analysis portion of this work is supported in part by the ONR award N00014-22-1-2813.]
Please include your amended statements within your cover letter; we will change the online submission form on your behalf."

We have removed the acknowledgments section and provided the updated funding statement in the cover letter.

[E: 6] "6. In the online submission form, you indicated that your data will be submitted to a repository upon acceptance. We strongly recommend all authors deposit their data before acceptance, as the process can be lengthy and hold up publication timelines. Please note that, though access restrictions are acceptable now, your entire minimal dataset will need to be made freely accessible if your manuscript is accepted for publication. This policy applies to all data except where public deposition would breach compliance with the protocol approved by your research ethics board. If you are unable to adhere to our open data policy, please kindly revise your statement to explain your reasoning and we will seek the editor's input on an exemption."

We have shared the data which is available at `https://github.com/piyushgupta221/Decision_making_in_ToH`.

Comments by Reviewer # 1

[R1: 1] "THIS PAPER CONNECTS RL TO EDUCATION. I'VE PERSONALLY BEEN LOOKING AT CONNECTING LLMS TO EDUCATION AND SO I ENJOYED SEEING THIS PAPER MAKE THESE CONNECTIONS. FROM MY CONVERSATIONS, THIS PAPER ADDRESSES A "HOT TOPIC" OF MATHEMATICAL THINKING IN EDUCATION. "

We're grateful that the reviewer found our manuscript engaging and relevant to an important topic.

[R1: 2] "THE STATISTICS AND EXPERIMENTS ARE CLEARLY DISCUSSED. PERSONALLY I LIKE USING BONFERRONI LIKE CONDITIONS FOR THE MODEL SELECTION (SAY RIC, OR UNBIASED ESTIMATES OF RISK) BUT THE METHODS USED ARE FINE FOR MAKING THE POINT FOR THE PAPER."

We're pleased that the reviewer found the statistics and experiments thoroughly discussed. While we acknowledge that Bonferroni-like conditions for model selection could be considered a viable alternative, we beleieve that the methods employed in our study are adequate to substantiate the claims presented in the paper.

Comments by Reviewer # 2

[R2: 1] "THANKS FOR INVITING ME TO COMMENT ON THE MANUSCRIPT "FOSTERING HUMAN LEARNING IN SEQUENTIAL DECISION-MAKING: UNDERSTANDING THE ROLE OF EVALUATIVE FEEDBACK". I AM AN EXPERT IN HUMAN DECISION MAKING IN THE CONTEXT OF AGENT/INDIVIDUAL BASED SIMULATION ECOLOGICAL AND ECONOMIC MODELS. I AM NOT FAMILIAR WITH THE "TOWER OF HANOI" GAME AND ITS SUBSTANTIAL LITERATURE. I AM ALSO NOT FAMILIAR "THE APPLICATION OF MAXIMUM ENTROPY IRL FOR LEARNING HUMAN REWARDS". THEREFORE, I WILL NOT COMMENT ON THE "MAXIMUM ENTROPY IRL" PART. I THINK AUTHORS SHOULD REFER TO "MAXIMUM ENTROPY IRL" ALREADY IN THE ABSTRACT. "

We thank the reviewer for their comment. In accordance with the reviewer's comment, we have referred to maximum entropy IRL in the abstract.

In the revised manuscript, we have added the following line in the abstract:

"Furthermore, treating the humans as noisy optimal agents, we employ maximum entropy inverse reinforcement learning to study the effect of feedback on the implicit human reward structure that explains their decision making."

[R2: 2] " SUMMARY OF THE PARTS THAT I ADDRESS IN MY REVIEW: THE AUTHORS STUDY THE "INFLUENCE OF EVALUATIVE FEEDBACK ON HUMAN DECISION-MAKING IN SEQUENTIAL TASKS.". THEREFORE, PARTICIPANTS SOLVED THE "TOWER OF HANOI" PUZZLE IN "FIVE SEPARATE EXPERIMENTS, EACH FEATURING A DIFFERENT TYPE OF FEEDBACK." "EACH PARTICIPANT FIRST SOLVED A 4-DISK TOH TASK TEN TIMES (TRAINING TASK) AND THEN A 5-DISK TOH TASK FIVE TIMES (TRANSFER TASK) TO EVALUATE THEIR SKILL TRANSFER TO A MORE CHALLENGING TASK." "

We thank the reviewer for their efforts and summarizing our work.

[R2: 3] "I THINK THE EXPERIMENT COULD BE BETTER EXPLAINED. MOST OF MY COMMENTS BELOW ARE ABOUT THE EXPERIMENT. MAYBE A SCREENSHOT IN THE APPENDIX WOULD HELP TO HAVE A BETTER INTUITION OF THE EXPERIMENT. WAS THERE A TIME CONSTRAIN? WAS TIME MEASURED AT ALL? AND IF SO DID IT CORRELATE WITH THE SUCCESS?"

We have added a screenshot of the training task in Experiment 5 and highlighted the differences in the other experiments with respect to the screenshot.

For each task, participants faced no time constraints; however, the maximum allowed moves were restricted to $m_{\text{allowed}} = \lceil 1.5 \times m_{\text{min}} \rceil$. Additionally, there was an overall time limit of 90 minutes allocated

for completing all the training and transfer tasks, and it's noteworthy that all participants successfully completed their tasks within this stipulated time frame.

In every trial, we recorded the time participants took for each move. However, we found no significant correlation between average reaction time and success rate. This suggests that success in the Tower of Hanoi is primarily dictated by mastering its inherent recursive patterns, rather than the duration of individual moves.

In the revised manuscript, we have added Fig. 3 and added the following:

"Fig. 3 shows the experimental interface utilized by participants during the training task of Experiment 5. As illustrated in Fig. 3, the participants had access to the numeric feedback, the number of moves taken, the current score S (total reward), the maximum available moves $m_{allowed}$, the maximum possible reward, as well as information regarding intermediate and final goal configurations.

The interface for the training tasks in the other experiments is similar to Experiment 5 with the following key differences with respect to Fig. 3:

(a) Experiment 1: Participants do not receive any numeric feedback, like "Bad Move: -2 (in Fig. 3)", and intermediate goal configurations during the training tasks.

(b) Experiment 2: Participants receive numeric feedback during training tasks, but no intermediate goal configurations are provided.

(c) Experiment 3: Participants were given access to a button labeled "Get Feedback" during the training tasks. Numeric feedback is only provided upon user request by pressing the designated button.

(d) Experiment 4: Participants receive intermediate goal configurations during training tasks, but no numeric feedback is provided.

"

" While we do not impose specific time limits for individual tasks, there was an overall time limit of 90 minutes allocated for completing all the training and transfer tasks. It's worth mentioning that all participants successfully completed their tasks within this stipulated time frame. "

[R2: 4] "THE FEEDBACK THAT PARTICIPANTS RECEIVED WHILE PLAYING IS OFTEN CALLED AI-DRIVEN – IS THAT APPROPRIATE FOR THE GIVEN EXAMPLE? I HAVE NOT REALLY THOUGHT ABOUT IT, BUT TO ME CONTRASTING THE STATE OF THE PLAYER WITH THE OPTIMAL PATH IS A SIMPLE CALCULATION? I MAY HAVE MISSED SOMETHING COMPLETELY HERE. IF SO THE AUTHORS MAY WANT TO MAKE SOME LESS TECHNICAL STATEMENTS FOR THE BROAD READERSHIP OF PLOS ONE. MAYBE MY SCHOOL OF THOUGHT IS ALSO A BIT RESTRICTIVE IN IDENTIFYING AI MAINLY WITH DEEP NEURAL NETWORKS."

The value function for each state in the ToH problem is proportional to the shortest path length to the target state, allowing for the application of simpler graph-search algorithms rather than RL. However, it's crucial to recognize that this characteristic is unique to ToH's finite and structured state space, governed by a recursive pattern, and does not apply to all sequential decision-making problems. In complex sequential decision-making problems like chess, characterized by larger or continuous state and action spaces, simpler algorithms might not be available, necessitating the use of advanced AI techniques like RL or deep neural networks to obtain the optimal policy. However, our framework is broadly applicable and can be generalized to other complex sequential decision-making tasks.

In the revised manuscript, we add the following line and Remark 1:

"We selected the Tower of Hanoi (ToH) puzzle as our choice for the sequential decision-making task. This choice is motivated by the simplicity of the ToH task, enabling efficient learning and evaluation within a reasonable timeframe for our experiment. Nonetheless, it's essential to note that the framework discussed

in our work is broadly applicable and can be generalized to other complex sequential decision-making tasks."

" Remark 1: The value function for each state in the ToH problem is proportional to the shortest path length to the target state, allowing for the application of simpler graph-search algorithms rather than RL. However, it's crucial to recognize that this characteristic is unique to ToH's finite and structured state space, governed by a recursive pattern, and does not apply to all sequential decision-making problems. In complex sequential decision-making problems like chess, characterized by larger or continuous state and action spaces, simpler algorithms might not be available, necessitating the use of advanced AI techniques like RL or deep neural networks to obtain the optimal policy. However, our framework is broadly applicable and can be generalized to other complex sequential decision-making tasks."

[R2: 5] "IS THE SAMPLE OF PARTICIPANTS UNBIASED GIVEN THE ENTRY CONDITIONS: "COMPLETED A MINIMUM OF 500 PRIOR STUDIES AND MAINTAINING A 98% APPROVAL RATE"."

Participants are randomly recruited in an unbiased manner through the AMT platform, with no collection of private information about them. Additionally, recruited participants have no prior experience with the ToH. The entry criteria, requiring a minimum of 500 prior studies and a 98% approval rate, are standard practice in online experiments. These criteria ensure higher data quality by selecting participants with a history of active engagement in experiments. By imposing such conditions, non-serious or non-engaging participants, who may not follow instructions carefully or interact with the experiment, are discouraged from participating.

[R2: 6] "THE AUTHORS PROVIDE NO DISCUSSION. MAYBE THIS IS NOT TO BE EXPECTED IN THE SPECIFIC RESEARCH DOMAIN? OR IS THE DISCUSSION MERGED IN THE OTHER PARTS – PLEASE COMMENT OR REVISE."

A detailed discussion of each result is included alongside the results in Sec $4.1 - 4.3$. To clarify this, we renamed Section 4 as 'Results and Discussion' and added another subsection, 4.4, to discuss the broad implications of our results.

[R2: 7] " SPECIFIC COMMENTS:
(A) PLEASE EXPLAIN ABBREVIATIONS AT THEIR FIRST OCCURRENCE, E.G. STEM, IOT (INTERNET OF THINGS), RL AGENT...
(B) THE ABSTRACT SHOULD CONTAIN THE KEY RESULTS OF THE STUDY.
(C) "3 HUMAN EXPERIMENTS": PLEASE PROVIDE NUMBERS FOR $m_{\min}$ AND $m_{\max}$ TO ALLOW INTUITION FOR THE CONDITION THE PARTICIPANTS HAVE BEEN. DID PARTICIPANTS KNOW THE RESTRICTION IN THE NUMBER OF ALLOWED MOVES.
(D) IS THE SCORE S VISIBLE FOR THE PARTICIPANTS?
(E) THE RANGE OF S IS DIFFERENT FOR EACH EXPERIMENT?
(F)LINE: 246 – HOW IS "VALUE OF THE STATE" DEFINED – MAYBE IT HAS BEEN STATED BEFORE, BUT IT WOULD BE HELPFUL TO READ IT HERE AGAIN.
(G)"3.2 METHODS": THAT MEANS N = 160 AND 32 PARTICIPANTS FOR EACH EXPERIMENT? OR WERE PARTICIPANTS SPREAD HETEROGENEOUSLY BETWEEN EXPERIMENTS?
(H)LINES 289 – O.K. HERE IS PART OF THE ANSWER GIVEN BY THE AUTHORS "WE COLLECT THE DATA OF 20 PARTICIPANTS EACH FOR THE 5 SET OF EXPERIMENTS"– WHAT HAPPENED WITH THE REMAINING 60 PARTICIPANTS?
(I) "RESULTS" – "PERCENTAGE SCORE" IS NOT COMPUTED USING THE SCORES S FROM SECTION "3 HUMAN EXPERIMENTS" – "PERCENTAGE SCORE" MAY HAVE VALUES BETWEEN 100 AND 12 ASSUMING $m_{\min} = 15$ AND $m_{used} \leq m_{allowed} = 1.5 * m_{\min}$... I GUESS $S_{percentage}$ IS SET TO ZERO AGAIN IF THE PARTICIPANTS FAIL TO SOLVE THE PUZZLE? STILL THE VISUALISATION BY BOXPLOTS IS NOT IDEAL, BECAUSE THEY SUGGEST THAT THE RESPONSE IS CONTINUOUS.
(J) FIGURE 6: THE AUTHORS SHOULD EXPLAIN THE COLOR CODING IN THE CAPTION OR ADD A LABEL TO THE LEGEND. "

(A) In the revised manuscript, we have explained all the abbreviations at their first occurrence.
(B) In the revised manuscript, we have added the key results in the abstract:

"Our findings underscore that humans perceive evaluative feedback as indicative of their long-term strategic success, thus aiding in skill acquisition and transfer in sequential decision-making tasks. Moreover, we demonstrate that evaluative feedback fosters a more structured and organized learning experience compared to learning without feedback. Furthermore, our results indicate that providing intermediate goals alone does not significantly enhance human learning outcomes."
(C) The minimum number of moves $m_{\min}$, is determined by the shortest distance from the initial to the final configuration in the state space of the Tower of Hanoi (ToH), as illustrated in Fig. 2. Since the final configuration is randomized, $m_{\min}$ varies for each experiment.

The maximum allowed moves m_{allowed}, for each configuration were calculated as $m_{\text{allowed}} = \lceil 1.5 \times m_{\min} \rceil$, as defined in equation (6).

The participants knew the restrictions on the number of allowed moves. The experimental interface, as depicted in Fig. 3, displayed to participants the maximum allowed moves and instructed them to solve the problem in as few steps as possible.

(D) Yes, the total accumulated score S is visible to the participants at all times and is updated after every move. However, a major component of the score $10(m_{\text{allowed}} - m_{\text{used}} + 1)$ depends on the number of moves used by the participant to solve the puzzle and is computed at the end of the experiment. The participants receive the final updated score at the end of each experiment. Nonetheless, the participants were informed about the scoring metric as a part of the experimental instructions.

(E) Yes, the range of S is different for each experiment and depends on the configuration of ToH and type of feedback. For each experiment, the participant can see the maximum possible score which can be achieved by solving the puzzle in minimum number of steps. In all our analysis, we utilize the percentage score $(0 - 100\%)$ given by equation (12) that is independent of the configuration and the type of feedback employed in the experiment.

(F) We have formally defined the value function in Section 2.1. Additionally, we have added the following recall statement in the revised manuscript.

"recall that the value of a state is proportional to the minimum path length of that state to the target state"

(G) The total number of participants is 160. Initially, we conducted Experiments 1 through 5, with 20 participants each, and the results are detailed in Section 4.1 - Performance under evaluative feedback.

As discussed in Section 4.1, additional data collection was undertaken for Experiments 1, 2, and 5, each with 20 participants, to facilitate the IRL analysis outlined in Sections 4.2 and 4.3. Notably, IRL analysis was not performed for Experiments 3 and 4, as the optional feedback (Experiment 3) and the sub-goal (Experiment 4) did not yield performance improvements for the participants.

(H) Refer to the previous response (G). The additional 60 participants data was collected for the IRL analysis in Sections 4.2 and 4.3.

(I) The scores in S are only for training tasks to allow the learning for participants. The evaluated percentage score used for the analysis and plots is independent of the type of feedback used. The range of the percentage score is $0 - 100\%$, where the maximum 100% is achieved when $m_{used} = m_{\min}$. The minimum 0% is achieved when the puzzle is still unsolved after $m_{used} = m_{allowed}$ and m_{used} transitions to $m_{allowed} + 1$. The minimum non-zero percentage score of $100 \times \frac{1}{m_{\text{allowed}} - m_{\min} + 1}$ is received by the participant when a puzzle is solved exactly after $m_{used} = m_{allowed}$ number of moves. While m_{allowed}, $m_{\min}$, and m_{used} are discrete parameters, their values are contingent upon the experimental setup, allowing for a diverse range of percentage scores spanning from 0% to 100%.

Due to the wide range of values the percentage score can assume, we employ box plots for visualization. Box plots are adept at representing discrete random variables, effectively illustrating key quantiles and extrema in the data distribution.

In the revised manuscript, we added the following:

"The maximum percentage score of 100% is achieved when $m_{used} = m_{\min}$. The minimum 0% is achieved when the puzzle is still unsolved after $m_{used} = m_{allowed}$ and m_{used} transitions to $m_{allowed} + 1$. The minimum non-zero percentage score of $100 \times \frac{1}{m_{\text{allowed}} - m_{\min} + 1}$ is received by the participant when a puzzle is solved exactly after $m_{used} = m_{allowed}$ number of moves. Since m_{allowed}, $m_{\min}$, and m_{used} are discrete parameters but depend on the experimental setup, the percentage score can take a large number of discrete values between 0% and 100%. Therefore, we employ box plots for visualization for effectively illustrating key quantiles and extrema in the data distribution."

(J) The colormap for each plot is already shown on the right of each plot. The red color represents high rewards close to 1 and dark blue represents close to 0 reward. In the revised manuscript, we update the image captions.
